# Dynamics of a modified Leslie–Gower predator–prey model with Holling-type II schemes and a prey refuge

**DOI:** 10.1186/s40064-016-2087-7

**Published:** 2016-04-14

**Authors:** Qin Yue

**Affiliations:** College of Finance and Mathematics, West Anhui University, Liuan, 237000 Anhui People’s Republic of China

**Keywords:** Leslie–Gower, Equilibrium, Global attractivity, Iterative, Refuge

## Abstract

We propose a modified Leslie–Gower predator–prey model with Holling-type II schemes and a prey refuge. The structure of equilibria and their linearized stability is investigated. By using the iterative technique and further precise analysis, sufficient conditions on the global attractivity of a positive equilibrium are obtained. Our results not only supplement but also improve some existing ones. Numerical simulations show the feasibility of our results.

## Background

The dynamic relationship between predators and their preys has long been and will continue to be one of the dominant themes in both ecology and mathematical ecology due to its universal existence and importance. Leslie (1948, 1958) introduced the following two species Leslie–Gower predator–prey model:

1 $$\left\{ \begin{array}{l} {\dot{x}}(t)=(r_1-b_1x)x-p(x)y,\\ {\dot{y}}(t)=\left( r_2- \frac{a_2 y}{x}\right) y,\\ \end{array} \right.$$

where *x*(*t*), *y*(*t*) stand for the population (the density) of the prey and the predator at time *t*, respectively. The parameters *r*_1_ and *r*_2_ are the intrinsic growth rates of the prey and the predator, respectively. *b*_1_ measures the strength of competition among individuals of species *x*. The value $\frac{r_1}{b_1}$ is the carrying capacity of the prey in the absence of predation. The predator consumes the prey according to the functional response *p*(*x*) and grows logistically with growth rate *r*_2_ and carrying capacity $\frac{r_2x}{a_2}$ proportional to the population size of the prey (or prey abundance). The parameter *a*_2_ is a measure of the food quantity that the prey provides and converted to predator birth. The term *y*/*x* is the Leslie–Gower term which measures the loss in the predator population due to rarity (per capita *y*/*x*) of its favorite food. Leslie model is a predator–prey model where the carrying capacity of the predator is proportional to the number of prey, stressing the fact that there are upper limits to the rates of increase in both prey *x* and predator *y*, which are not recognized in the Lotka–Volterra model.

As was pointed out by Aziz-Alaoui and Daher (2003), in the case of severe scarcity, *y* can switch over to other populations but its growth will be limited by the fact that its most favorite food *x* is not available in abundance. In order to solve such deficiency in system (1), Aziz-Alaoui and Daher (2003) proposed and studied the following predator–prey model with modified Leslie–Gower and Holling-type II schemes:

2 $$\left\{ \begin{array}{l} {\dot{x}}(t) =\left( r_1-b_1x- \frac{a_1y}{x+k_1}\right) x,\\ {\dot{y}}(t) = \left( r_2- \frac{a_2y}{x+k_2}\right) y, \end{array} \right.$$

where *r*_1_, *b*_1_, *r*_2_, *a*_2_ have the samemeaning as in system (1). *a*_1_ is the maximum value which per capita reduction rate of *x* can attain; *k*_1_ and *k*_2_ measure the extent to which environment provides protection to prey *x* and to predator *y* respectively. They obtained the boundedness and global stability of positive equilibrium of system (1). Since then, many scholars considered system (2) and its non-autonomous versions by incorporating delay, impulses, harvesting, stochastic perturbation and so on (see, for example, Yu 2012; Nindjin et al. 2006; Yafia et al. 2007, 2008; Nindjin and Aziz-Alaoui 2008; Gakkhar and Singh 2006; Guo and Song 2008; Song and Li 2008; Zhu and Wang 2011; Liu and Wang 2013; Kar and Ghorai 2011; Huo et al. 2011; Li et al. 2012; Liu et al. 2013; Gupta and Chandra 2013; Ji et al. 2009, 2011; Yu 2014; Yu and Chen 2014; Yue 2015). In particular, Yu (2012) studied the structure, linearized stability and the global asymptotic stability of equilibria of (2) and obtained the following result (see Theorem 3.1 in Yu 2012):

### **Theorem 1**

*Assume that*

$$\begin{aligned} \begin{array}{ll} a_1r_1r_2+a_1b_1r_2k_2<a_2b_1r_1k_1,&\quad (C_1)\\ 2a_2b_1M+(a_2b_1k_1-a_2r_1-a_1r_2)>0,&\quad (C_2) \end{array} \end{aligned}$$

*hold, where*$M=\frac{r_1k_1-a_1L}{b_1k_1}$*and*$L=\frac{r_1r_2+b_1r_2k_2}{a_2b_1}$*, then system* (2) *has a unique positive equilibrium which is globally attractive.*

As was pointed out by Kar (2005), mite predator–prey interactions often exhibit spatial refugia which afford the prey some degree of protection from predation and reduce the chance of extinction due to predation. In Kar (2005), Tapan Kumar Kar had considered a predator–prey model with Holling type II response function and a prey refuge. The author obtained conditions on persistent criteria and stability of the equilibria and limit cycle for the system. For more works on this direction, one could refer to Kar (2005), Srinivasu and Gayatri (2005), Ko and Ryu (2006), Huang et al. (2006), Kar (2006), González-Olivares and Ramos-Jiliberto (2003), Ma et al. (2009), Chen et al. (2009, 2010, 2012), Ji and Wu (2010), Tao et al. (2011) and the references cited therein.

Although many authors have considered the dynamic behaviors of the modified Leslie–Gower model (Yu 2012; Nindjin et al. 2006; Yafia et al. 2007, 2008; Nindjin and Aziz-Alaoui 2008; Gakkhar and Singh 2006; Guo and Song 2008; Song and Li 2008; Zhu and Wang 2011; Liu and Wang 2013; Kar and Ghorai 2011; Huo et al. 2011; Li et al. 2012; Liu et al. 2013; Gupta and Chandra 2013; Ji et al. 2009, 2011; Yu 2014; Yu and Chen 2014; Yue 2015) and predator–prey with a prey refuge (Kar 2005; Srinivasu and Gayatri 2005; Ko and Ryu 2006; Huang et al. 2006; Kar 2006; González-Olivares and Ramos-Jiliberto 2003; Ma et al. 2009; Chen et al. 2009, 2010, 2012; Ji and Wu 2010; Tao et al. 2011), as far as we know, there are almost no literatures discussing the modified Leslie–Gower model with a prey refuge. Stimulated by the works of Kar (2005), Srinivasu and Gayatri (2005), Ko and Ryu (2006), Huang et al. (2006), Kar (2006), González-Olivares and Ramos-Jiliberto (2003), Ma et al. (2009), Chen et al. (2009, 2010, 2012), Ji and Wu (2010), Tao et al. (2011), we will extend model (2) by incorporating a refuge protecting *mx* of the prey, where $m\in [0,1)$ is constant. This leaves $(1-m)x$ of the prey available to the predator, and modifying system (2) accordingly to the system:

3 $$\begin{aligned} \left\{ \begin{array}{l} {\dot{x}}(t) = \left( r_1-b_1x- \frac{a_1(1-m)y}{(1-m)x+k_1}\right) x,\\ {\dot{y}}(t) = \left( r_2- \frac{a_2y}{(1-m)x+k_2}\right) y. \end{array} \right. \end{aligned}$$

system (2) is the special case of (3) with *m* = 0, i.e. there is no prey refuge. By using iterative technique and further precise analysis, we finally obtain the following main results:

### **Theorem 2**

*Suppose that*

$$a_1(1-m)^2r_1r_2+a_1(1-m)b_1r_2k_2<a_2b_1r_1k_1,\qquad (C_3)$$

*holds, then system* (3) *has a unique positive equilibrium*$(x^*,y^*)$*which is globally attractive.*

Theorem 2 shows that $\lim \nolimits _{t\rightarrow \infty } x(t)=x^*, \lim \nolimits _{t\rightarrow \infty } y(t)=y^*$. Notice that $x^*$ and $y^*$ are only dependent with the coefficients of system (3), and independent of the solution of system (3). Thus we can get the following result:

### **Corollary 1**

*Suppose thatC*_3_*holds, then system* (2) *is permanent.*

When *m* = 0 that is there is no prey refuge, (3) becomes to (2) and *C*_3_ becomes to *C*_1_, so as a direct corollary of Theorem 2, we have:

### **Corollary 2**

*Suppose thatC*_1_*holds, then system* (2) *has a unique positive equilibrium which is globally attractive.*

Comparing with Theorem 1, it follows from Corollary 2 that *C*_2_ is superfluous, so our results improve the main results in Yu (2012). Moreover, when consider the case of no alternate prey, so $k_2=0$ (this is often called the Holling-Tanner model), by the similar proof of Theorem 2, we can obtain:

### **Corollary 3**

*Suppose that*

$$a_1(1-m)^2r_1r_2<a_2b_1r_1k_1,\qquad (C_4)$$

*holds, then system* (3) *with*$k_2=0$*has a unique positive equilibrium*$(x^*,y^*)$*which is globally attractive.*

The remaining part of this paper is organized as follows. In section “Nonnegative equilibria and their linearized stability”, we discuss the structure of nonnegative equilibria to (3) and their linearized stability. We prove the main result (i.e. Theorem 2) of this paper in section “Global attractivity of a positive equilibrium”. Then, in section “Examples and numeric simulations”, a suitable example together with its numeric simulations is given to illustrate the feasibility of the main results. We end this paper by a briefly discussion.

## Nonnegative equilibria and their linearized stability

Obviously, (3) has three boundary equilibria, $E_0=(0,0)$, $E_1=( \frac{r_1}{b_1},0)$, and $E_2=\left( 0, \frac{r_2k_2}{a_2}\right)$. Set $B\triangleq a_1r_2(1-m)^2-a_2r_1(1-m)+a_2b_1k_1$ and $\Delta \triangleq B^2-4(1-m)a_2b_1[(1-m)a_1r_2k_2-a_2r_1k_1]$. As for the existence of positive equilibria and linearized stability of equilibria, similar to the discussion in Yu (2012), we have the following results:

**Case 1.** Suppose one of the following conditions holds.

(i) $$m>1- \frac{a_2r_1k_1}{a_1r_2k_2}$$.
(ii) $$m=1- \frac{a_2r_1k_1}{a_1r_2k_2}\quad {\text{and}}\quad B<0.$$
(iii) $$m<1- \frac{a_2r_1k_1}{a_1r_2k_2},B<0,\quad {\text{and}}\quad \Delta =0.$$

Then (3) has a unique positive equilibrium $E_{3,1}=(x_{3,1}, y_{3,1})$ with $x_{3,1}=\frac{-B+\sqrt{\Delta }}{2(1-m)a_2b_1}$ and $y_{3,1}=\frac{r_2\left( (1-m)x_{3,1}+k_2\right) }{a_2}$.

**Case 2.** If $m<1-\frac{a_2r_1k_1}{a_1r_2k_2}$, *B* < 0, and $\Delta >0$, then (3) has two positive equilibria $E_{3,\pm }=(x_{3,\pm }, y_{3,\pm })$, where $x_{3,\pm }=\frac{-B\pm \sqrt{\Delta }}{2(1-m)a_2b_1}$ and $y_{3,\pm }=\frac{r_2\left( (1-m)x_{3,\pm }+k_2\right) }{a_2}$.

**Case 3.** If no condition in Case 1 or Case 2 holds, then (3) has no positive equilibrium.

### **Proposition 1**

(i): Both $E_0$ and $E_1$ are unstable.
(ii): $E_2$ is locally asymptotically stable if $m<1- \frac{a_2r_1k_1}{a_1r_2k_2}$ while it is unstable if $m>1- \frac{a_2r_1k_1}{a_1r_2k_2}$.
(iii): The positive equilibrium $E_{3,1}$ in Case 1(i)(ii) is stable if $2b_1(1-m)x_{3,1}^2-(r_1(1-m)-r_2(1-m)-b_1k_1)x_{3,1}+k_1r_2>0$.
(iv): The positive equilibrium $E_{3,-}$ is unstable while the positive equilibrium $E_{3,+}=(x_{3,+}, y_{3,+})$ is stable if $2b_1(1-m)x_{3,+}^2-(r_1(1-m)-r_2(1-m)-b_1k_1)x_{3,+}+k_1r_2>0$.

When *m* = 0 that is there is no prey refuge, Proposition 1 becomes to Propositions 2.1 and 2.2 in Yu (2012). Thus our results supplement the exist ones. In the coming section, we will prove the main result (i.e. Theorem 2) of this paper.

## Global attractivity of a positive equilibrium

In this section, we first introduce several lemmas which will be useful in proving the main result (i.e. Theorem 2) of this paper.

### **Lemma 1**

(Chen et al. 2007) *If*$a>0$, $b>0$*and*${\dot{x}}\ge x(b-ax)$*, when*$t\ge 0$*and*$x(0)>0$*, we have:*

$$\liminf \limits _{t\rightarrow +\infty }x(t)\ge \frac{b}{a}.$$

*If*$a>0$, $b>0$*and*${\dot{x}}\le x(b-ax)$*, when*$t\ge 0$*and*$x(0)>0$*, we have:*

$$\limsup \limits _{t\rightarrow +\infty }x(t)\le \frac{b}{a}.$$

Now, we prove the main result of this paper.

### *Proof of Theorem 2*

Let $(x(t),y(t))^T$ be any positive solution of (3). From condition $(C_3)$, we can choose a small enough $\varepsilon >0$ such that

4 $$\begin{aligned} & \frac{a_2b_1r_1k_1-a_1(1-m)^2r_1r_2-a_1(1-m)b_1r_2k_2}{a_2b_1k_1}\\ & \;\; -\left( \frac{a_1(1-m)^2r_2}{a_2k_1}+\frac{a_1(1-m)}{k_1}\right) \varepsilon >0 . \end{aligned}$$

The first equation of (3) yields

5 $${\dot{x}}(t)\le \left( r_1-b_1x\right) x.$$

By applying Lemma 1 to (5) leads to

$$\limsup \limits _{t\rightarrow +\infty }x(t)\le \frac{r_1}{ b_1}.$$

Hence, for above $\varepsilon >0$, there exists a $T_1 > 0$ such that

6 $$x(t)\le \frac{r_1}{ b_1}+\varepsilon \mathop {=}\limits ^{\mathrm {\bigtriangleup }}M_1^{(1)}.$$

(6) together with the second equation of (3) leads to

7 $${\dot{y}}(t)\le \left( r_2- \frac{a_2y}{(1-m)M_1^{(1)}+k_2}\right) y, \quad {\hbox {for all}}\quad t\ge T_1.$$

From (7), according to Lemma 1, we can obtain

$$\limsup \limits _{t\rightarrow +\infty }y(t)\le \frac{r_2\left( (1-m)M_1^{(1)}+k_2\right) }{ a_2}.$$

Thus, for above $\varepsilon$, there exists a $T_2\ge T_1,$ such that

8 $$y(t)\le \frac{r_2\left( (1-m)M_1^{(1)}+k_2\right) }{ a_2}+\varepsilon \mathop {=}\limits ^{\mathrm {\bigtriangleup }}M_{2}^{(1)},\quad {\hbox {for all}}\,\,t\ge T_2.$$

(8) together with the first equation of (3) leads to

9 $${\dot{x}}(t)\ge \left( r_1-b_1x-\frac{a_1(1-m)M_{2}^{(1)}}{k_1}\right) x,\quad {\hbox {for all}}\,\, t\ge T_2.$$

According to (4), we can obtain

10 $$\begin{aligned} r_1- \frac{a_1(1-m)M_{2}^{(1)}}{k_1}&= r_1- \frac{a_1(1-m)r_2}{a_2k_1}\left( \frac{(1-m)r_1}{b_1}+k_2\right) \\&\quad -\frac{a_1(1-m)}{k_1}\left( \frac{r_2(1-m)}{a_2}+1\right) \varepsilon \\&= \frac{a_2b_1r_1k_1-a_1(1-m)^2r_1r_2-a_1(1-m)b_1r_2k_2}{a_2b_1k_1} \\&\quad -\left( \frac{a_1(1-m)^2r_2}{a_2k_1}+\frac{a_1(1-m)}{k_1}\right) \varepsilon >0, \end{aligned}$$

Therefore, by Lemma 1 and (9), we have

$$\liminf \limits _{t\rightarrow +\infty }x(t)\ge \frac{r_1-\frac{a_1(1-m)M_{2}^{(1)}}{k_1}}{ b_1}.$$

Hence, for above $\varepsilon$, there exists a $T_3\ge T_2,$ such that

11 $$\begin{aligned} x(t)\ge \frac{r_1-\frac{a_1(1-m)M_{2}^{(1)}}{k_1}}{ b_1}-\varepsilon \mathop {=}\limits ^{\mathrm {\bigtriangleup }}m_{1}^{(1)},\quad {\hbox {for all}}\,\, t\ge T_3. \end{aligned}$$

From (11) and the second equation of system (3), we know that for $t\ge T_3,$

12 $${\dot{y}}(t)\ge \left( r_2-\frac{a_2y}{(1-m)m_{1}^{(1)}+k_2}\right) y.$$

Applying Lemma 1 to (12) leads to

$$\liminf \limits _{t\rightarrow +\infty }y(t)\ge \frac{r_2\left( (1-m)m_{1}^{(1)}+k_2^l\right) }{a_2}.$$

That is, for above $\varepsilon$, there exists a $T_4>T_3$ such that

13 $$y(t)\ge \frac{r_2\left( (1-m)m_{1}^{(1)}+k_2\right) }{a_2}-\varepsilon \mathop {=}\limits ^{\mathrm {\bigtriangleup }}m_{2}^{(1)},\quad {\hbox {for all}}\,\,t\ge T_4.$$

From (6), (8), (11) and (13), for $t\ge T_4$, we have

14 $$0<m_{1}^{(1)}\le x(t)\le M_{1}^{(1)},\quad 0<m_{2}^{(1)}\le y(t)\le M_{2}^{(1)}.$$

(14) together with the first equation of (3) leads to

$${\dot{x}}(t)\le \left( r_1-b_1x- \frac{a_1(1-m)m_{2}^{(1)}}{(1-m)M_{1}^{(1)}+k_1}\right) x,\quad {\hbox {for all}}\,\,t\ge T_4.$$

From (10) and (14), we have

$$\begin{aligned} r_1- \frac{a_1(1-m)m_{2}^{(1)}}{(1-m)M_{1}^{(1)}+k_1}>r_1- \frac{a_1(1-m)m_{2}^{(1)}}{k_1}\ge r_1- \frac{a_1(1-m)M_{2}^{(1)}}{k_1}>0. \end{aligned}$$

Therefore, similarly to the analysis of (5–6), for above $\varepsilon$, there exists a $T_5>T_4$ such that

15 $$x(t)\le \frac{r_1- \frac{a_1(1-m)m_{2}^{(1)}}{(1-m)M_{1}^{(1)}+k_1}}{ b_1}+ \frac{\varepsilon }{2}\mathop {=}\limits ^{\mathrm {\bigtriangleup }}M_1^{(2)}.$$

Compare (6) with (15), one can get

16 $$M_1^{(2)}<M_1^{(1)}.$$

Substituting (15) into the second equation of system (3), we have

17 $${\dot{y}}(t)\le \left( r_2- \frac{a_2y}{(1-m)M_1^{(2)}+k_2}\right) y, \quad {\hbox {for all}}\,\,t\ge T_5.$$

Applying Lemma 1 to the above inequality leads to

$$\limsup \limits _{t\rightarrow +\infty }y(t)\le \frac{r_2\left( (1-m)M_1^{(2)}+k_2\right) }{ a_2}.$$

Thus, for above $\varepsilon$, there exists a $T_6\ge T_5,$ such that

18 $$\begin{aligned} y(t)\le \frac{r_2\left( (1-m)M_1^{(2)}+k_2\right) }{ a_2}+ \frac{\varepsilon }{2}\mathop {=}\limits ^{\mathrm {\bigtriangleup }}M_{2}^{(2)},\quad {\hbox {for all}}\,\,t\ge T_6. \end{aligned}$$

From (8), (16) and (18) , we have

19 $$M_2^{(2)}<M_2^{(1)}.$$

Substituting (11) and (18) into the first equation of system (3), we obtain

$$\begin{aligned} {\dot{x}}(t)\ge \left( r_1-b_1x-\frac{a_1(1-m)M_{2}^{(2)}}{(1-m)m_1^{(1)}+k_1}\right) x,\quad {\hbox {for all}}\,\,t\ge T_6. \end{aligned}$$

According to (10) and (19), we have

$$\begin{aligned} r_1-\frac{a_1(1-m)M_{2}^{(2)}}{(1-m)m_1^{(1)}+k_1}>r_1- \frac{a_1(1-m)M_{2}^{(1)}}{k_1}>0 \end{aligned}$$

Thus, similarly to the above analysis, for above $\varepsilon$, there exists a $T_7\ge T_6,$ such that

20 $$\begin{aligned} x(t)\ge \frac{r_1-\frac{a_1(1-m)M_{2}^{(2)}}{(1-m)m_1^{(1)}+k_1}}{ b_1}- \frac{\varepsilon }{2}\mathop {=}\limits ^{\mathrm {\bigtriangleup }}m_{1}^{(2)},\quad {\hbox {for all}}\,\,t\ge T_7. \end{aligned}$$

From (11), (19) and (20) , we have

21 $$m_1^{(1)}<m_1^{(2)}.$$

It follows from (20) and the second equation of system (3) that

22 $$\begin{aligned} {\dot{y}}(t)\ge \left( r_2-\frac{a_2y}{(1-m)m_{1}^{(2)}+k_2}\right) y,\quad {\hbox {for all}}\,\,t\ge T_7. \end{aligned}$$

Thus, similarly to the above analysis, for above $\varepsilon$, there exists a $T_8\ge T_7,$ such that

23 $$\begin{aligned} y(t)\ge \frac{r_2\left( (1-m)m_{1}^{(2)}+k_2\right) }{a_2}- \frac{\varepsilon }{2}\mathop {=}\limits ^{\mathrm {\bigtriangleup }}m_{2}^{(2)},\quad {\hbox {for all}}\,\,t\ge T_8. \end{aligned}$$

From (13), (21) and (23) , we have

24 $$m_2^{(1)}<m_2^{(2)}.$$

Therefore, it follows from (14), (16), (19), (21) and (24) that

25 $$\begin{aligned} \begin{array}{ll} 0<m_{1}^{(1)}<m_{1}^{(2)}\le x(t)<M_{1}^{(2)}\le M_{1}^{(1)},&\\ 0<m_{2}^{(1)}<m_{2}^{(2)}\le y(t)\le M_{2}^{(2)}<M_{2}^{(1)},&\quad {\hbox {for all}}\,\,t\ge T_8. \end{array} \end{aligned}$$

Repeating the above procedure, we get four sequences $$M_i^{(n)},$$$$m_i^{(n)},$$$$i=1,2,\,n=1,2,\ldots$$, such that

26 $$\begin{aligned} M_1^{(n)}&= \frac{r_1- \frac{a_1(1-m)m_{2}^{(n-1)}}{(1-m)M_{1}^{(n-1)}+k_1}}{ b_1}+ \frac{\varepsilon }{n},\qquad M_{2}^{(n)}= \frac{r_2\left( (1-m)M_1^{(n)}+k_2\right) }{ a_2}+ \frac{\varepsilon }{n} \\ m_1^{(n)}&= \frac{r_1- \frac{a_1(1-m)M_{2}^{(n)}}{(1-m)m_{1}^{(n-1)}+k_1}}{ b_1}- \frac{\varepsilon }{n},\qquad m_{2}^{(n)}= \frac{r_2\left( (1-m)m_1^{(n)}+k_2\right) }{ a_2}- \frac{\varepsilon }{n} \end{aligned}$$

Now, We go to show that the sequences $M_i^{(n)}$ are non-increasing, and the sequences $m_i^{(n)}$, are non-decreasing for *i* = 1, 2 by induction. Firstly, from (25), we immediately get

$$M_i^{(2)}<M_i^{(1)},\,\,\,m_i^{(2)}>m_i^{(1)},\quad i=1,2.$$

Let us suppose that for n,

$$M_i^{(n)}<M_i^{(n-1)},\,\,\,m_i^{(n)}>m_i^{(n-1)},\quad i=1,2.$$

By direct computation, one can obtain

27 $$\begin{aligned} M_1^{(n+1)}&= \frac{r_1- \frac{a_1(1-m)m_{2}^{(n)}}{(1-m)M_{1}^{(n)}+k_1}}{ b_1}+ \frac{\varepsilon }{n+1}< \frac{r_1- \frac{a_1(1-m)m_{2}^{(n-1)}}{(1-m)M_{1}^{(n-1)}+k_1}}{ b_1}+ \frac{\varepsilon }{n}=M_1^{(n)} \\ M_{2}^{(n+1)}&= \frac{r_2\left( (1-m)M_1^{(n+1)}+k_2\right) }{ a_2}+ \frac{\varepsilon }{n+1}< \frac{r_2\left( (1-m)M_1^{(n)}+k_2\right) }{ a_2}+ \frac{\varepsilon }{n}=M_{2}^{(n)} \\ m_1^{(n+1)}&= \frac{r_1- \frac{a_1(1-m)M_{2}^{(n+1)}}{(1-m)m_{1}^{(n)}+k_1}}{ b_1}- \frac{\varepsilon }{n+1}< \frac{r_1- \frac{a_1(1-m)M_{2}^{(n)}}{(1-m)m_{1}^{(n-1)}+k_1}}{ b_1}- \frac{\varepsilon }{n}=m_1^{(n)} \\ m_{2}^{(n+1)}&= \frac{r_2\left( (1-m)m_1^{(n+1)}+k_2\right) }{ a_2}- \frac{\varepsilon }{n+1}< \frac{r_2\left( (1-m)m_1^{(n)}+k_2\right) }{ a_2}- \frac{\varepsilon }{n}=m_{2}^{(n)} \end{aligned}$$

Therefore, we have that

$$\begin{aligned} &0<m_1^{(1)}<m_1^{(2)}<\cdots<m_1^{(n)}<x(t)<M_1^{(n)}<\cdots<M_1^{(2)}<M_1^{(1)},\\ &0<m_2^{(1)}<m_2^{(2)}<\cdots<m_2^{(n)}<y(t)<M_2^{(n)}<\cdots<M_2^{(2)}<M_2^{(1)}, \end{aligned}$$

Hence, the limits of $M_i^{(n)}$ and $m_i^{(n)}$, $i=1,2$, $n=1,2,\ldots$ exist. Denote that

$$\begin{aligned} \lim \limits _{n\rightarrow +\infty }M_1^{(n)}={\overline{x}},\,\,\lim \limits _{n\rightarrow +\infty }m_1^{(n)}={\underline{x}},\,\,\lim \limits _{n\rightarrow +\infty }M_2^{(n)}={\overline{y}},\,\,\lim \limits _{n\rightarrow +\infty }m_2^{(n)}={\underline{y}}. \end{aligned}$$

Hence ${\overline{x}}\ge {\underline{x}},\,\,{\overline{y}}\ge {\underline{y}}.$ Letting $n\rightarrow +\infty$ in (26), we immediately

28 $$\begin{aligned}&r_1-b_1{\overline{x}}- \frac{a_1(1-m) {\underline{y}}}{(1-m){\overline{x}}+k_1}=0,\qquad r_2- \frac{a_2{\overline{y}}}{(1-m){\overline{x}}+k_2}=0 \\&r_1-b_1{\underline{x}}- \frac{a_1(1-m){\overline{y}}}{(1-m){\underline{x}}+k_1}=0,\qquad r_2- \frac{a_2{\underline{y}}}{(1-m){\underline{x}}+k_2}=0 \end{aligned}$$

It follows from (28) that

29 $$\begin{aligned} a_2(r_1-b_1{\overline{x}})\left( (1-m){\overline{x}}+k_1\right)&= a_1r_2(1-m)\left( (1-m){\underline{x}}+k_2\right) , \\ a_2(r_1-b_1{\underline{x}})\left( (1-m){\underline{x}}+k_1 \right)&= a_1r_2(1-m)\left( (1-m){\overline{x}}+k_2\right) . \end{aligned}$$

Multiplying the second equality of (29) by −1 and adding it to the first equality of (29), we have

$$({\overline{x}}-{\underline{x}})\left( a_1r_2(1-m)^2+a_2r_1(1-m)-a_2b_1k_1-a_2b_1(1-m)({\overline{x}}+{\underline{x}})\right) =0.$$

We claim ${\overline{x}}={\underline{x}}$. Otherwise, ${\overline{x}}\ne {\underline{x}}$ and

30 $$a_2b_1(1-m)({\overline{x}}+{\underline{x}})=a_1r_2(1-m)^2+a_2r_1(1-m)-a_2b_1k_1$$

Substituting (30) into (29), we have

$$\begin{aligned} a_2^2b_1(r_1-b_1{\overline{x}})((1-m){\overline{x}}+k_1)&= a_1r_2(1-m)(a_1r_2(1-m)^2+a_2r_1(1-m)\\&\quad -a_2b_1k_1+a_2b_1k_2-a_2b_1(1-m){\overline{x}}),\\ a_2^2b_1(r_1-b_1{\underline{x}})((1-m){\underline{x}}+k_1)&= a_1r_2(1-m)(a_1r_2(1-m)^2+a_2r_1(1-m)\\&\quad -a_2b_1k_1+a_2b_1k_2-a_2b_1(1-m){\underline{x}}). \end{aligned}$$

Thus, ${\overline{x}}$ and ${\underline{x}}$ are two positive solutions of the following equation

31 $$\begin{aligned} a_2^2b_1(r_1-b_1x)((1-m)x+k_1)&= a_1r_2(1-m)(a_1r_2(1-m)^2+a_2r_1(1-m) \\&\quad-a_2b_1k_1+a_2b_1k_2-a_2b_1(1-m)x). \end{aligned}$$

Simplifying (31), one can get

32 $$a_2^2b_1^2(1-m)x^2+a_2b_1(a_2b_1k_1-a_2r_1(1-m)-a_1r_2(1-m)^2)x+D=0,$$

where $D=a_2(a_1(1-m)^2r_1r_2+a_1(1-m)b_1r_2k_2-a_2b_1r_1k_1)+a_1r_2(1-m)(a_1r_2(1-m)^2-a_2b_1k_1).$ (*H*1) shows that $a_1(1-m)^2r_1r_2+a_1(1-m)b_1r_2k_2-a_2b_1r_1k_1<0$ and $a_1r_2(1-m)^2-a_2b_1k_1<0$. Hence, *D* < 0, that is, Eq. (31) does not have two positive solutions. So ${\overline{x}}={\underline{x}}$ and consequently, ${\overline{y}}={\underline{y}}$. Obviously, *C*_3_ implies $a_1(1-m)r_2k_2<a_2r_1k_1$ or $m>1- \frac{a_2r_1k_1}{a_1r_2k_2}$, that is, condition (i) of Case 1 holds. Thus (3) has a unique positive equilibrium $(x^*,y^*)$ and $(x^*,y^*)$ also satisfies (28). Therefor ${\overline{x}}={\underline{x}}=x^*$ and ${\overline{y}}={\underline{y}}=y^*$, that is to say

$$\lim \limits _{t\rightarrow \infty } x(t)=x^*,\quad \lim \limits _{t\rightarrow \infty } y(t)=y^*.$$

and this completes the proof. $\square$

## Examples and numeric simulations

Consider the following example:

33 $$\begin{aligned} {\dot{x}}&= \left( 11-5x- \frac{2.4y}{0.6x+6.5}\right) x, \\ {\dot{y}}& = \left( 8- \frac{2y}{0.6x+2}\right) y. \end{aligned}$$

In this case, we have $r_1=11, b_1=5, a_1=4, m=0.4, k_1=6.5, r_2=8, a_2=2, k_2=2$ and $B=a_1r_2(1-m)^2-a_2r_1(1-m)+a_2b_1k_1=63.32, \Delta =B^2-4(1-m)a_2b_1[(1-m)a_1r_2k_2-a_2r_1k_1]=6519.8,{\text{so}}$

$$\begin{aligned}x^*=\frac{-B+\sqrt{\Delta }}{2(1-m)a_2b_1}\approx 1.4521,\quad y^*=\frac{r_2\left( (1-m)x^*+k_2\right) }{a_2}\approx 11.485. \end{aligned}$$

By simple computation, we also have

$$a_1(1-m)^2r_1r_2+a_1(1-m)b_1r_2k_2-a_2b_1r_1k_1=-396.28<0.$$

**Fig. 1:**
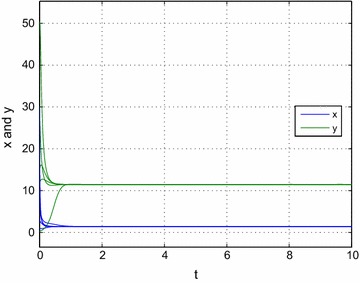
Dynamic behavior of the system (33) with the initial condition (*x*(0), *y*(0)) = (3, 12)^*T*^, (1, 30)^*T*^, (10, 0.3)^*T*^, (8, 15)^*T*^ and (30, 50)^*T*^, respectively

Thus, conditions in Theorem 2 are satisfied, hence, system (33) has a unique positive equilibrium $E^*=(x^*,y^*)$ which is globally attractive. Numerical simulation also confirms our result (see Fig. 1).

## Conclusion

In this paper, we consider a modified Leslie–Gower predator–prey model with Holling-type II schemes and a prey refuge. The structure of equilibria and their linearized stability is investigated. Morever, by using the iterative technique and further precise analysis, sufficient conditions on the global attractivity of a positive equilibrium are obtained. When *m* = 0 that is there is no prey refuge, (3) we discussed reduces to (2) which was studied by Yu (2012). Yu (2012) have provided a sufficient condition on the global asymptotic stability of a positive equilibrium by employing the Fluctuation Lemma and obtained Theorem 1. By comparing Theorems 1 with Corollary 2, we find that the condition *C*_2_ in Theorem 1 is redundant. Thus our results not only supplement but also improve some existing ones. The numerical simulation of system (33) verify our main results. It follows from Theorem 2 and condition *C*_3_ that increasing the amount of refuge can ensure the coexistence and attractivity of the two species more easily. This is rational, since the existence of alternate prey can prevent the predator from extinction and increasing the amount of refuge could protect more prey from predation and become permanent. Note that for the diffusion/PDE model where refuge can be spatial, whether refuge can change global attractivity of the interior equilibrium? This is a further problem, which can be studied in the future.
